# Academic Well-Being Among STEM University Students Living Away from Home: A Mixed-Methods Study

**DOI:** 10.3390/ijerph23050608

**Published:** 2026-05-04

**Authors:** Barbara Loera, Federica Graziano, Giorgia Molinengo, Daniela Converso, Giulia Bacci

**Affiliations:** Department of Psychology, University of Turin, 10124 Torino, Italy; barbara.loera@unito.it (B.L.); daniela.converso@unito.it (D.C.); giulia.bacci@unito.it (G.B.)

**Keywords:** academic well-being, engagement, burnout, student self-efficacy, university, mixed-methods, thematic analysis, regression analysis, non-resident students

## Abstract

**Highlights:**

**Public health relevance—How does this work relate to a public health issue?**

- University student mental health, particularly in high-demand STEM environments, represents a growing public health concern linked to burnout, disengagement, and reduced academic persistence.
- Students living away from home and international students face additional structural and social demands that may increase vulnerability to emotional exhaustion and reduced academic engagement.

**Public health significance—Why is this work of significance to public health?**

- By integrating qualitative narratives with quantitative indicators, this study identifies specific institutional and pedagogical factors systematically associated with burnout, engagement, and academic functioning.
- Teaching-related experiences emerged as the most consistent correlate of psychological outcomes, highlighting the role of organizational contexts in shaping student well-being.

**Public health implications—What are the key implications or messages for practitioners, policy makers and/or researchers in public health?**

- Structural and modifiable aspects of university environments—such as teaching practices, workload organization, and communication systems—represent actionable targets for mental health promotion strategies in higher education.
- Mixed-methods approaches can inform institution-level prevention frameworks by linking lived experiences to measurable psychological outcomes.

**Abstract:**

Background: University students’ mental health represents an increasing public health concern, particularly in STEM contexts characterized by high academic demands. Students living away from home, including international students, may face additional stressors related to relocation, social integration, and adaptation. This study examined how narrated academic experiences are associated with psychological and academic functioning among relocated STEM students. Methods: A cross-sectional convergent parallel mixed-methods study was conducted at an Italian STEM university (May–June 2024). An online survey was distributed to the entire accessible student population (33,336 invitations; 12,538 accesses; response rate = 37.6%). Analyses focused on relocated students who completed all relevant sections (*N* = 776; M age = 22.96). Quantitative measures assessed academic self-efficacy, burnout (Emotional Exhaustion; Cynicism), engagement (Vigor; Dedication), study program satisfaction, and perceived academic goal attainment. Open-ended responses underwent thematic analysis with a codebook approach and transformed into category count variables. Hierarchical regression models examined associations controlling for age, gender, and academic level. Results: Organizational and learning-related difficulties were the most frequent categories. Content categories explained additional variance across outcomes (ΔR^2^ = 0.054–0.107). Teaching-related narratives were associated with higher burnout and lower engagement and satisfaction, whereas Positive narratives showed the opposite pattern. Conclusions: Institutional and pedagogical experiences are systematically associated with student well-being among relocated STEM students, highlighting modifiable targets for university-level mental health promotion strategies.

## 1. Introduction

The transition to university represents a critical developmental milestone in young adulthood, characterised by significant academic, social, and psychological challenges [1]. These challenges are especially evident in Science, Technology, Engineering, and Mathematics (STEM) institutions, which are distinguished by their rigorous curricula and exacting performance standards. In such contexts, the combination of intensive workloads and structured assessment systems can lead to an increased vulnerability to stress and psychological strain [2,3].

Recent global evidence indicates that mental health difficulties among university students have reached concerning levels [4]. A growing body of research has recently focused on the issue of academic burnout, which is defined as a syndrome characterised by emotional exhaustion related to study demands, cynicism towards academic tasks, and reduced academic efficacy [5]. Burnout has been identified as a significant issue within STEM programmes, with research indicating that workload and assessment structures contribute to sustained academic pressure [6]. These symptoms have been associated with diminished academic performance, heightened intentions to drop out, and a deterioration in mental health outcomes [7].

Within the Job Demands–Resources (JD-R) model, burnout arises when demands exceed available resources [8]. In STEM universities, demands may include complex coursework, laboratory commitments, compressed examination periods, and performance-based evaluation systems. Conversely, academic resources such as supportive teaching practices, transparent assessment criteria, constructive feedback, autonomy-supportive learning environments, and social support foster academic engagement, defined as a positive state of vigour, dedication, and absorption in study activities [5,9]. Engagement has consistently been linked to greater well-being, persistence, and academic success.

Vulnerability to burnout and disengagement may be heightened among specific student groups, particularly those living away from home (non-commuter students) and international students. Relocating introduces additional non-academic demands, including housing, financial strain, time management, and rebuilding social networks. For international students, these demands are further compounded by language barriers, cultural adaptation, bureaucratic complexity, and potential experiences of discrimination or social exclusion [10].

Research has shown that reduced social integration and sense of belonging are associated with higher burnout and lower engagement [2]. This is particularly relevant for students living away from home and international students, who must establish new relationships while navigating a challenging academic environment. Moreover, literature on the “chilly climate” in STEM disciplines suggests that women and minority students may experience lower belonging, exposure to subtle bias, and reduced academic self-efficacy [11,12], factors that may further compromise well-being in these contexts.

Teaching and learning environments play a crucial mediating role in this dynamic. Supportive and transparent teaching practices and practical learning approaches are associated with higher engagement and lower burnout risk [9]. Conversely, excessive academic workload, perceived unfairness in evaluation, limited exam opportunities, organisational disarray, and ineffective communication may act as structural stressors within the JD-R framework. Recent extensions of the JD-R framework to student populations further support its relevance in explaining academic burnout and engagement processes [13].

Despite growing research on burnout and engagement in higher education, limited evidence simultaneously considers the STEM context, students living away from home, and the integration of qualitative and quantitative data. Research in Southern European contexts also remains limited.

This study addresses this by integrating quantitative and qualitative data to examine academic well-being among STEM students living away from home, including international students. In line with a convergent parallel mixed-methods design, qualitative data are systematically transformed into quantitative indicators to enable the examination of associations between narrated experiences and psychological outcomes, while preserving their interpretive grounding. Using this mixed-methods approach, the research examines indicators of psychological well-being, academic burnout, and academic engagement, alongside students’ lived experiences. By situating well-being within the structural, pedagogical, and residential ecology of a STEM institution, this study aims to identify both critical stressors and protective resources that shape the university experience.

Research Questions:

1. How are narrated academic experiences associated with burnout, engagement, and academic functioning among STEM students living away from home?
2. Which qualitative content categories are most strongly associated with psychological outcomes?
3. To what extent do institutional and pedagogical factors represent modifiable determinants of student well-being?

## 2. Materials and Methods

### 2.1. Procedure and Participants

The present study adopted a cross-sectional mixed-methods design integrating quantitative survey measures with qualitative thematic analysis. A convergent parallel approach was implemented, whereby qualitative data derived from open-ended responses were systematically transformed into quantitative variables to enable statistical integration.

Data were collected between May and June 2024 through an online survey administered via the institutional platform to university students enrolled in STEM programs at an Italian university. Participants were invited through official institutional communication channels and accessed the survey via a QR code. Participation was voluntary, anonymous, and uncompensated. All respondents provided informed consent in accordance with EU General Data Protection Regulation (2016/679). The study adhered to the principles of the Declaration of Helsinki and complied with all relevant ethical guidelines for research involving human participants. The present study is part of a broader institutional research project on psychological well-being in academia, commissioned by the Rector and formally approved by the University’s Board of Administration as well as by the academic and administrative staff union representatives.

A total of 33,336 invitations were distributed, and 12,538 students accessed the survey (response rate = 37.6%). Of these, 7793 completed only the quantitative section, whereas 1561 completed the full survey including open-ended responses. The present study focuses on a subsample of 776 participants who (a) completed all relevant sections of the questionnaire and (b) had relocated to attend university, living in student residences or shared private apartments. This analytic subsample was selected to ensure conceptual coherence with the study aims. These students did not benefit from a pre-existing familial or local social support network in the university city, rendering them a theoretically relevant subgroup for examining the relationship between perceived academic experiences and psychological functioning.

The final sample consisted of 776 university students (M = 22.96, SD = 3.21; range 18–49). Most participants were male (66.9%), while 30.8% were female. Regarding academic level, 56.8% were enrolled in a Bachelor’s degree program and 43.2% in a Master’s degree program. Most participants (90.3%) were Italian students who completed the open-ended responses in Italian, while the remaining percentage consisted of international students who responded in English.

### 2.2. Measures

The questionnaire was structured into three sections.

The first section collected essential sociodemographic and academic information, including gender identity, age, academic level, year of enrolment, degree program, residential status (in-town vs. relocated), and nationality (assessed dichotomously as Italian/non-Italian to minimize intrusiveness).

The second section included validated measures assessing key dimensions of academic functioning and psychological well-being, specifically academic self-efficacy, burnout, engagement, study program satisfaction, and perceived academic goal attainment.

Academic self-efficacy was assessed using 10 items rated on a 4-point Likert scale ranging from 0 (*Not at all*) to 3 (*Completely*). Items evaluated students’ perceived capability to successfully manage academic demands, including learning complex content, maintaining motivation, and achieving study goals. The scale is conceptually grounded in social-cognitive theory and aligned with previously validated measures of student self-efficacy [14]. Item scores were summed to obtain a total score (range 0–30), with higher scores indicating greater academic self-efficacy.

Academic burnout was measured using items adapted from the Maslach Burnout Inventory-Student Survey (MBI-SS [5,15]. Items were rated on a 7-point frequency scale ranging from 0 (*Never*) to 6 (*Always*). Consistent with the core conceptualization of student burnout, only the two core dimensions—Emotional Exhaustion (5 items; range 0–30) and Cynicism (4 items; range 0–24)—were included in the present analyses. Higher scores reflect greater burnout.

Academic engagement was assessed using items consistent with the Utrecht Work Engagement Scale–Student version (UWES-S) [5]. Items were rated on the same 7-point frequency scale (0 = *Never*; 6 = *Always*). In line with the theoretical core dimensions of engagement, Vigor (5 items; range 0–30) and Dedication (5 items; range 0–30) were considered in the present study. Higher scores indicate higher academic engagement.

Study program satisfaction was assessed using a single item asking participants to rate their overall satisfaction with their academic pathway on a scale from 1 (*Not at all satisfied*) to 10 (*Completely satisfied*).

Perceived academic goal attainment was measured using a single item asking students to estimate the percentage of their academic goals achieved since the beginning of the academic year (0–100%). Higher scores indicate greater perceived progress toward educational objectives.

The third section of the questionnaire comprised an open-ended question allowing participants to freely report perceptions, experiences, and suggestions regarding their academic life and well-being.

### 2.3. Statistical Analysis

First, a thematic analysis of answers to open-ended questions was performed following a codebook approach [16,17]) and using Atlas.ti 25 software. The analysis was carried out in multiple phases:

1. Data preparation: all texts were anonymized by removing names or other references that could make them recognizable in any way. All documents were translated in English by a professional translator and accurately revised by the research team to check for the accuracy of the content after translation.
2. First round of coding on a subset of documents (*n* = 137): The initial round of coding was carried out by one of the researchers. Due to the large amount of textual data, the AI-assisted Coding procedure provided by Atlas.ti was used as an initial support tool to facilitate code generation to be used as a starting point for subsequent data interpretation. This first round produced a large amount of codes/subcodes that were systematically and iteratively reviewed in a process of constant discussion with a second researcher to ensure methodological rigor. Codes were eliminated/discarded when irrelevant, merged when overlapping, renamed and iteratively refined and aggregated into overarching categories to align with the study aims. As indicated in codebook approaches to thematic analysis, divergences were addressed through discussion until consensus was reached. This collaborative approach ensured transparency and rigor. Moreover, AI-generated suggestions remained always subordinate to the researcher’s choices [18,19].
3. Second round of coding on all documents (*N* = 776): the final coding system was applied on the whole textual corpus. Again, this iterative process was carried out by one of the researchers in a process of constant discussion with a second one. Discrepancies were resolved through discussion. In this phase, some codes were refined and others added to capture additional nuances in meanings. The results of the final coding were discussed and approved by the research team.

To integrate qualitative findings with quantitative psychological outcomes, a quantizing approach was adopted within a mixed-methods framework. This approach involves transforming qualitative data into numerical variables to enable statistical analysis while preserving their interpretive grounding in participants’ narratives.

The rationale for this transformation is rooted in convergent parallel mixed-methods designs, where qualitative and quantitative data are collected simultaneously and integrated at the analysis stage to provide complementary insights into the same phenomenon [20,21]. In this context, converting qualitative categories into quantitative indicators allows the systematic examination of associations between narrated experiences and psychological outcomes.

Importantly, this transformation does not aim to reduce qualitative data to purely numerical form, but to enable cross-case comparability and to identify patterns of co-occurrence between experiential domains and standardized measures. This procedure is consistent with established mixed-methods approaches that use quantized data to bridge interpretive depth and analytic generalizability [20].

Identified codes were transformed into dichotomous variables (0 = absent; 1 = present). Codes were subsequently grouped into theoretically defined categories (Communication problems, Economic concerns, Future work concerns, Gender issues, Health concerns, International students’ difficulties, Learning, Organizational practical difficulties, Special needs, Psychological impact, Social relationships, Teaching, and Positive feelings) (Table 1). Categories were developed iteratively by the two researchers involved in the process of coding and discussed with the research team. For each participant, a category count score was computed within each category by summing codes.

As for quantitative analysis, internal consistency was first assessed for all multi-item scales using Cronbach’s alpha (α) and McDonald’s omega (ω).

Integration of qualitative and quantitative data occurred at the analysis stage: content category scores were entered as predictors in hierarchical multiple regression models alongside standardized psychological measures. These models were conducted to examine associations between content categories and students’ psychological and academic functioning, including Emotional Exhaustion, Cynicism, Vigor, Dedication, Self-Efficacy, Study Program Satisfaction, and Perceived Academic Goal Attainment. Consistent with the theoretical framework, only the core dimensions of burnout (Emotional Exhaustion and Cynicism) and engagement (Vigor and Dedication) were included in the analyses.

For each outcome, regression analyses were conducted in two steps. In Model 1, sociodemographic covariates were entered, including age (continuous), gender (female = 1, male = 0), and academic level (Bachelor’s degree student = 1, Master’s degree student= 0). In Model 2, content category count variables were simultaneously added to examine their additional explanatory contribution beyond sociodemographic factors. All regression coefficients reported in the final models are standardized (β) to allow comparability across predictors. The incremental variance explained by content categories was evaluated using ΔR^2^ (change in R^2^ between Model 1 and Model 2).

Given the cross-sectional design, results were interpreted as associative rather than causal. In line with the transactional mixed-methods framework adopted in this study, analyses were intended to explore reciprocal patterns of co-occurrence between thematic salience in students’ narratives and psychological and academic functioning, without assuming directional effects.

Statistical analyses were performed using IBM SPSS Statistics (Version 30).

## 3. Results

### 3.1. Content Categories

The 13 key categories developed from the analysis of the students’ spontaneous comments encompass a range of perspectives observed across the student dataset (Table 1). These categories reflected various perceptions of the factors influencing the well-being of non-local and international students at the university under review. For some categories, results are presented with reference to codes to provide more detailed information on the content of quotations. To facilitate interpretation, the content categories are presented by mapping institutional and study-related needs (such as workload and organisational constraints) and resources (such as supportive teaching and positive experiences). These are then associated with burnout, commitment, and academic performance in the context of the JD-R model.

Category 1. Communication problems.

The category of “communication problems,” with its 17 occurrences, highlights recurring difficulties students face in obtaining clear and timely information from administration and faculty, due to contradictory messages, inaccessible channels, and prolonged response times. This lack of transparency is perceived not merely as a practical inconvenience, but as a deficiency in care and consideration toward students, intensifying feelings of disorientation and abandonment—particularly during critical academic moments such as enrolment, examinations, and administrative deadlines—and ultimately undermining trust in the institution.

*“What I think would be a good improvement is having information about applying to this University and each step of your studies clearly presented, to orient yourself in all the information is extremely unclear and time consuming. It would be great to know what are the requirements and processes in advance.”*

[Participant 754]

International students in the sample also encounter these difficulties, both with the relevant offices and with professors.

*“…and also I think communication of professors and students should be much more than now…cause it will be better effects on us.”*

[Participant 241]

Category 2. Economic concerns.

The category of “economic concerns,” with its 24 occurrences, highlights a structural and pervasive challenge for students living away from home, for whom financial sustainability is essential. High housing costs, often exceeding scholarships, combined with daily expenses, make financial stability precarious. This situation leads students to perceive access to education as increasingly dependent on economic means, with limited support measures excluding many eligible individuals. As a result, economic insecurity shapes the entire university experience, constraining choices, social opportunities, and even the continuation of studies, ultimately undermining motivation and well-being.

*“Ensuring more services for those who have a high ISEE (Equivalent Economic Situation Indicator) but which does not actually reflect the true economic situation of the family.”*

[Participant 235]

It has been reported by a significant number of international students that they encounter difficulties in locating reasonably priced accommodation.

*“When I arrived here, I had a hard time finding a house because there is no rental house for foreign students, and asking for an Italian guarantor is very strange for someone who just arrived here and is even far from my parents.”*

[Participant 405]

Category 3. Future work concerns.

The category of “future work concerns,” with its 14 occurrences, reflects a significant sense of uncertainty among students living away from home, whose educational paths often entail substantial sacrifices. Students express doubts about the value of their qualifications and their alignment with labour market demands, alongside concerns about the need for further relocation or the inability to secure employment. These anxieties extend beyond employability to include job location and conditions, and are reinforced by perceptions of a labour market marked by slow career progression, limited merit recognition, and stagnant wages.

*“It would be nice if there were more information about possible career opportunities for their master’s degree course. For example, what roles they can fill, what they consist of, what salaries to expect, and which ones are at the lower end of the scale.”*

[Participant 543]

*“Invite engineers/architects to present the different professional roles that exist, because after 5 years of study we still have no clear idea of the opportunities we could have.”*

[Participant 606]

Category 4. Health concerns.

The category of “health concerns,” with its 11 occurrences, highlights challenges faced by students living away from home in managing their physical well-being. Difficulties in accessing healthcare services and the absence of family support increase the risk of neglecting health issues. Although less frequently mentioned, this aspect significantly affects students’ quality of life and academic performance, underscoring an often overlooked dimension of well-being.

*“Last but not least, it is essential to improve academic medical support by, for example, creating a “University Medical Clinic” with medical staff available for general consultations and preventive services.”*

[Participant 66]

Category 5. Gender issues.

The category of “gender issues,” with its 21 occurrences, highlights the often invisible challenges faced by female students living away from home in STEM contexts. Students report the need to constantly prove their competence in unwelcoming environments, experiencing interruptions, disregard of their contributions, and, in some cases, harassment. These dynamics reveal a context that can range from subtly exclusionary to openly hostile.

*“There are still some professors (of “advanced” age and men) who sometimes make comments that can be understood as “offensive” towards the entire female gender. They are really very few, I want to emphasize, but they are there (as an order of magnitude, one professor in my entire master’s degree course).”*

[Participant 397]

*“Harassment by students makes life very difficult for a small minority, but the inability of professors to transmit effectively makes life difficult for everyone, males, females, or any member of the LGBTQI+ spectrum.”*

[Participant 483]

Category 6. International students’ difficulties.

The category of “international students’ difficulties,” with its 29 occurrences, highlights the multifaceted challenges faced by international students, including general academic and bureaucratic issues, as well as difficulties adapting to a new university system. Additional barriers include isolation, cultural and dietary adjustment, and language obstacles affecting both academic participation and social life. Instances of discrimination (3 occurrences) and racism (3 occurrences), though less frequent, underscore the presence of exclusionary dynamics within the academic environment.

*“What I think is we students come so far from our houses for studying here and few teachers are unfair with us because of the ethnicity. In my opinion they should be educated about the equality and be aware about that the students who came here to adopt the culture should encourage us in adopting not discouraging.”*

[Participant 164; discrimination, racism]

*“Focus more on international students. As an International student I feel alone I don’t have Italian friends. They speak Italian all the time. I unable to communicate.”*

[Participant 257; isolation, language barrier]

Category 7. Learning.

The category of “learning,” with its 399 occurrences, emerges as a central factor for student well-being, encompassing pedagogical, structural, and relational dimensions. Students call for more engaging and practical teaching, while highlighting issues in course organisation and limited peer collaboration. Two key structural concerns stand out: excessive academic workload, leading to chronic fatigue, and insufficient exam sessions, which concentrate assessments into short periods and significantly increase stress.

*“For example, exemptions or mid-terms for subjects could be introduced to allow students to subdivide the study best and facilitate them in passing the exam itself. Besides lightening the study load for us students, in my opinion, it would also facilitate professors and raise the exam pass percentages.”*

[Participants 480; academic workload, lack of exam session]

*“Make it more practical based learning rather than just theory.”*

[Participant 140, practical learning]

*“Recorded video lessons and the possibility of taking partial exams can be a great help in studying, because during sessions I often feel anxious about the exams I have to take in a limited period of time.”*

[Participant 107, class organization; exam anxiety]

Category 8. Teaching.

The category of “teaching,” with its 206 occurrences, is a key area affecting student well-being, particularly in relation to teaching methods and assessment practices. Students report dissatisfaction with instructional quality, lack of engagement, and misalignment with their learning needs. Assessment emerges as the main critical issue, with concerns about transparency, fairness, and coherence with actual knowledge, often generating anxiety. Additional issues, such as perceived teacher fatigue and unfair treatment, further contribute to a broader perception of inadequacy in teaching practices.

*“In the courses I have attended, almost all professors still believe they are living in a bygone era, the period in which they attended university. Students are not listened to or understood; we are simply a student number. The typical professor at this university is tyrannical, authoritarian, touchy, haughty and never humble. Before becoming professors, they should take a course on how to treat students, on how to relate to people who live in a different historical period from the one in which the professor was educated and grew up. ENOUGH WITH PEOPLE FULL OF EGO FOR THE ACHIEVEMENTS THEY HAVE MADE! BEFORE BEING STUDENTS, WE ARE PEOPLE, OR RATHER YOUNG PEOPLE. OUR PROFESSORS DO NOT TEACH HOW TO LIVE IN AN EQUAL SOCIETY, BUT TEACH STUDENTS TO BE SUBMISSIVE.”*

[Participant 6, authority issues, competition, critique of teaching methods]

Category 9. Organisational practical difficulties.

The prevalence of the category “Organisational practical difficulties” (412 occurrences) indicates the most widespread barrier to student well-being, profoundly impacting daily academic life. These encompass inadequate access to psychological support services (accessibility psychological service), deficiencies in catering provisions (food and canteen concerns), housing issues, and perceived disorganisation in administrative and communication processes.

Two specific codes exert significant influence: time management, a persistent challenge in balancing academic, personal, and professional commitments; and campus other spaces, referring to non-teaching areas (study spaces, social areas, relaxation zones) that are notably inadequate, underscoring an unmet need for functional and welcoming environments.

*“Have more places to study, the study rooms are always full and the free classrooms, the few that rightly exist, are used by students to chat and very often there is so much noise that you can’t study. Every time I look for a place to study, I lose at least an hour because, as I just wrote, it’s difficult to find a quiet place to study peacefully and, above all, with sockets to use the PC (many classrooms don’t even have sockets near the desks).”*

[Participant 80, campus other spaces; disorganization]

*“Like most of my classmates, I found the second term to be challenging from an academic point of view. There were five courses, each requiring weekly assignments. This created a rather unsustainable pace. I decided not to take one of the courses and to take the exam when it is available. For a student living away from home, it is a real challenge, but it is also a form of personal growth. I would recommend, in my humble opinion, better organisation of the first year, perhaps by moving a more theoretical exam from the second term to the first.”*

[Participant 48, time management; housing issue]

Category 10. Special needs.

Although the category of Special needs is addressed in only five quotations, it highlights a crucial concern for the well-being of university students: the existence of specific learning disorders (SLD), regardless of whether they have been formally diagnosed. The low number of occurrences should not be misleading; it may reflect not a reduced incidence of the phenomenon, but rather students’ difficulty in recognising, articulating, or reporting their need for support.

*“There are not enough tools provided to allow neurodivergent people to complete their academic path peacefully. A neurodivergent person struggles to attend lectures and, at least in my case, I found no compensatory tools—such as recorded lessons—that could help me. I ended up not attending classes and preparing exams entirely from home on my own with whatever material I could find; however, this is a major source of stress, discomfort, and unhappiness for me.”*

[Participant 592]

Category 11. Psychological impact.

The category of Psychological impact, with 175 occurrences, represents the most significant emotional and psychological dimension of the university experience, reflecting a widespread sense of unease among students. The manifestations of this malaise are varied and interconnected, including lack of motivation, pervasive anxiety, feelings of helplessness (7 occurrences), and profound hopelessness. While the need to maintain a healthy work–life balance is clear, three particularly salient codes dominate the narrative. Stress is the most immediate and widespread response to academic pressures, while frustration reflects the sense of helplessness in the face of systemic and personal obstacles. Additionally, mental health is explicitly mentioned by students, indicating a growing awareness of their own psychological well-being. References to suicidal thoughts (2 occurrences) and depression (6 occurrences), though numerically limited, serve as an unequivocal wake-up call, highlighting the need for targeted and accessible support interventions.

*“(…) Walking through the corridors of the polytechnic is like crossing a circle of hell, where you see souls in torment, curved and sad, wandering aimlessly. I leave the Polytechnic with the idea and feeling that I have wasted the last six years of my life, as I do not feel at all like a grown-up compared to when I enrolled. I believe that my mental stability has gradually diminished. I too have become a sad person, lacking enthusiasm and incapable of feeling joy or pleasure.”*

[Participant 546; depression, frustration, mental health]

Category 12. Social relationships.

The category of “social relationships,” with its 53 occurrences, underscores a key dimension of student well-being, marked by a strong need for socialisation and dedicated spaces for interaction. At the same time, social isolation, accounting for 27 occurrences, emerges as the most significant issue, reflecting a widespread difficulty in building meaningful relationships within a fragmented and weakly community-oriented academic environment.

*“Create recreational spaces where students can relax between classes. These spaces can also be an opportunity to meet new people, especially for first-year students and those from out of town who have just arrived in this city and don’t yet have a group of friends. Not everyone is outgoing enough to approach classmates in the lecture hall; in these recreational spaces, activities (such as games) could be organized to help overcome shyness and allow students to make new friends.”*

[Participant 588, socialization spaces]

Category 13. Positive feelings.

Although the Positive feelings category includes only 28 quotations, compared to the substantial number of critical issues identified in other areas, it offers significant insight into the factors contributing to university students’ well-being. The image is marked by a pervasive sense of satisfaction, which, with 21 instances, emerges as the most significant code. Participants express gratitude for positive educational experiences, the achievement of personal goals, and the recognition of their commitment. Additionally, feelings of gratitude towards teachers or colleagues, the perception of a welcoming and stimulating environment (2), and a more general sense of personal well-being (1) also emerge, though less frequently.

*“In my opinion, this university is already doing everything possible to help the students’ experience. Such as using anonymous questionnaires, having staff on hand to help in case of difficulties, *etc.*”*

[Participant 5; satisfaction]

### 3.2. Categories Co-Occurrence Analysis

**Figure 1:**
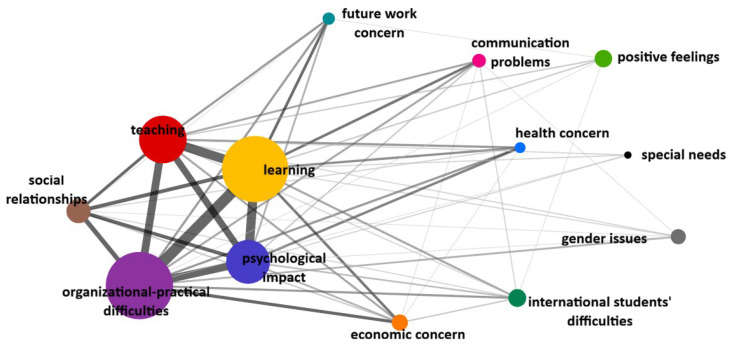
Graphical representation of categories co-occurrences in the total sample. Note: The size of the circles indicates the number of occurrences of categories, circles that are closer and connected by thick lines indicate stronger co-occurrences between categories.

The analysis of co-occurrences between categories illustrates not only the relevance of each category, but also the relationships between them and their degree of closeness or proximity. This analysis allows for a deeper understanding of the qualitative results and gives useful suggestions for the interpretation of the following quantitative analysis. As seen above, the largest categories were Organisational practical difficulties and Learning, followed by Teaching and Psychological impact. These categories are closely interconnected (co-occurrences between Learning and Organisational practical difficulties *n* = 172; Learning and Psychological impact *n* = 121; Learning and Teaching *n* = 121; Psychological impact and organisational practical difficulties *n* = 86; Psychological impact and Teaching *n* = 66) (Figure 1).

The category of Social relationships is smaller but related to Organisational practical difficulties (co-occurrences *n* = 31), Learning (co-occurrences *n* = 21), and Psychological impact (co-occurrences *n* = 19).

The categories related to Communication problems, Health, Future work, and Economic concerns are positioned around this central core. In particular, the strongest co-occurrences are between Communication problems and Learning (*n* = 10); Economic concerns and both Organisational practical difficulties (*n* = 14) and Learning (*n* = 10); Future work concern and Learning (*n* = 11). Co-occurrences between Health concerns and both Psychological impact (*n* = 9) and Learning (*n* = 8) are weaker but remarkable.

In a more peripheral position, but co-occurring with the central core, are the categories of International students’ concerns, Gender issues, Special needs and Positive Feelings.

The categories’ co-occurrence analysis was carried out in subgroups of participants in relation to sex (male/female), degree level (bachelor/master), and geographical origin (Italian/international students) to explore potential specific patterns.

**Figure 2:**
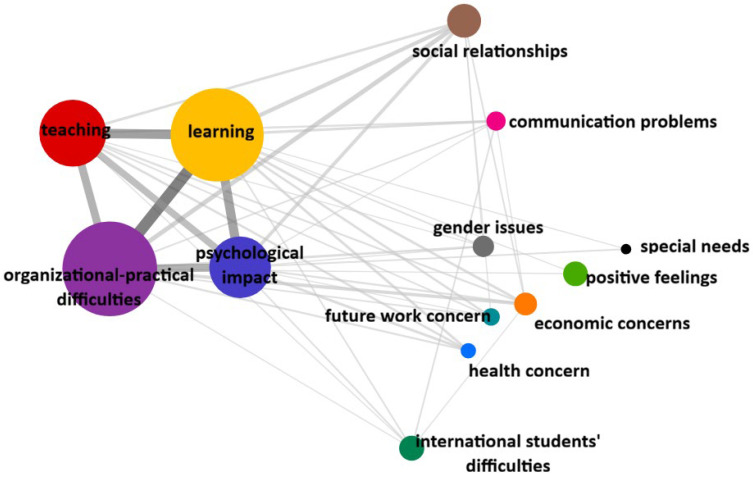
Graphical representation of Categories co-occurrence in the subgroup of Bachelor’s degree students. Note: The size of the circles indicates the number of occurrences of categories, circles that are closer and connected by thick lines indicate stronger co-occurrences between categories.

**Figure 3:**
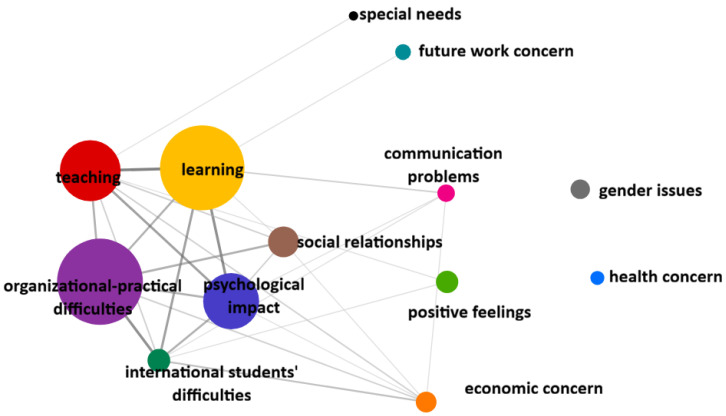
Graphical representation of category co-occurrences in the subgroup of international students. Note: The size of the circles indicates the number of occurrences of categories, circles that are closer and connected by thick lines indicate stronger co-occurrences between categories.

Compared to the total sample, different patterns emerge when considering the subgroups of Bachelor’s degree students (Figure 2) and international students (Figure 3).

In the subgroup of Bachelor’s degree students (Figure 2), the core was always made up of the four categories, namely Organisational practical difficulties, Learning, Teaching and Psychological impact; all the other aspects, albeit with varying degrees of relevance, appeared to be equidistant from the core. In particular, the category of Social relationships was relevant, but the co-occurrence with the central core was weaker than in the total sample (co-occurrences with Learning *n* = 12; with Organisational practical difficulties *n* = 15; with Psychological impact *n* = 9; with Teaching *n* = 5). The same is true for the Economic concerns (co-occurrences with Learning *n* = 5; with organisational practical difficulties *n* = 8; with Psychological impact *n* = 3; with Teaching *n* = 3) and Future work concerns (co-occurrences with Learning *n* = 4; with Organisational practical difficulties *n* = 2; with Psychological impact *n* = 1; with Teaching *n* = 2).

Considering the subgroup of international students (Figure 3), the core is dominated by Organisational practical difficulties and Learning, followed by Teaching and Psychological impact; the categories that most co-occur with the core are Social relationships (co-occurrences with Learning *n* = 1; with Organisational practical difficulties *n* = 4; with Psychological impact *n* = 2; with Teaching *n* = 2) and International students’ difficulties (co-occurrences with Learning *n* = 5; with organisational practical difficulties *n* = 6; with Psychological impact *n* = 4; with Teaching *n* = 2). In a more peripheral position, but co-occurring with the central core, are the categories of Communication problems and Economic concerns, and Positive Feelings; more distal is the position of the other two categories, namely Future work concerns and Special needs. Finally, the categories of Health concerns and Gender issues are distant and disconnected from the others, indicating issues that are present but probably highly specific to some individuals.

### 3.3. Reliability and Intercorrelations Among Study Variables

All multi-item scales demonstrated excellent internal consistency. Cronbach’s alpha ranged from 0.84 to 0.93 and McDonald’s omega ranged from 0.90 to 0.95 across subscales. Descriptive statistics for all outcome variables are reported in Table 2.

Correlation analyses revealed theoretically coherent patterns. Burnout dimensions were negatively associated with engagement, self-efficacy, study program satisfaction, and perceived academic goal attainment, whereas engagement and self-efficacy were positively interrelated and positively associated with indicators of academic functioning. These patterns provide additional support for the expected convergent and discriminant validity of the measures used in the present study, including the coherent functioning of the two single-item indicators.

### 3.4. Connecting Codes with Scores

Hierarchical regression analyses (Table 3) showed that content categories accounted for a meaningful proportion of variance beyond sociodemographic covariates. In Model 1, gender, age and academic level were associated with a relatively small proportion of variance across outcomes (R^2^ ranging from 0.006 to 0.034). However, academic level showed a consistent pattern: being enrolled in a Bachelor’s degree program was negatively associated with self-efficacy (β = −0.121, *p* < 0.01) and perceived academic goal attainment (β = −0.161, *p* < 0.001), indicating lower perceived competence and reduced perceived academic success compared to more advanced students. This pattern suggests that earlier stages of the academic pathway may be characterized by greater perceived difficulty and lower subjective academic consolidation.

In contrast, gender was not significantly associated with most psychological or academic outcomes. Despite the STEM context of the university, no systematic gender differences emerged in burnout, engagement, self-efficacy, satisfaction, or perceived goal attainment, suggesting comparable psychological functioning across male and female students within this sample.

The inclusion of content category scores in Model 2 was associated with a consistent increase in variance accounted for across all dependent variables. The incremental variance (ΔR^2^) ranged from 0.054 (Dedication) to 0.107 (Study Program Satisfaction), with particularly notable increases for Vigor (ΔR^2^ = 0.091), Self-Efficacy (ΔR^2^ = 0.074), and Emotional Exhaustion (ΔR^2^ = 0.061).

Overall, full models accounted for between 6.3% (Dedication) and 13.1% (Study Program Satisfaction) of variance in the outcomes, indicating that content categories contribute relevant associative information beyond demographic factors.

Among content categories, Teaching and Positive feelings emerged as the most consistent and robust correlates across outcomes. Teaching was positively associated with burnout dimensions (Emotional Exhaustion and Cynicism) and negatively associated with engagement (Vigor and Dedication), self-efficacy, study program satisfaction, and perceived academic goal attainment. Notably, Teaching showed the strongest negative association with Study Program Satisfaction (β = −0.222, *p* < 0.001) and Dedication (β = −0.153, *p* < 0.001), indicating a systematic pattern linking critical academic-organizational experiences to lower psychological and academic functioning.

Conversely, Positive feelings demonstrated an opposite pattern. They were negatively associated with burnout and positively associated with engagement, self-efficacy, study program satisfaction, and perceived academic goal attainment. The strongest association emerged for Vigor (β = 0.233, *p* < 0.001), followed by Study Program Satisfaction (β = 0.162, *p* < 0.001) and Self-Efficacy (β = 0.149, *p* < 0.001), suggesting that positive narrative content co-occurs with higher psychological energy and academic adjustment.

Beyond Teaching and Positive feelings, several other categories showed significant associations with specific outcomes. Learning was positively associated with Emotional Exhaustion (β = 0.092, *p* < 0.05) and Cynicism (β = 0.094, *p* < 0.05), and negatively associated with Dedication (β = −0.074, *p* < 0.05) and Self-Efficacy (β = −0.099, *p* < 0.01). This pattern suggests that narratives emphasizing academic workload, organization of courses, or exam-related concerns tend to co-occur with higher burnout and lower academic confidence and involvement.

The psychological impact category was positively associated with Emotional Exhaustion (β = 0.120, *p* < 0.001) and Cynicism (β = 0.083, *p* < 0.05), and negatively associated with Study Program Satisfaction (β = −0.077, *p* < 0.05), denoting that narratives focused on stress, anxiety, or emotional strain are systematically linked with higher psychological fatigue and reduced academic satisfaction.

International students’ difficulties category showed a differentiated pattern. They were positively associated with Vigor (β = 0.081, *p* < 0.05) but negatively associated with Perceived Academic Goal Attainment (β = −0.098, *p* < 0.01). This suggests a more complex configuration, in which references to international or adaptation-related challenges co-occur with both higher engagement energy and lower perceived academic progress.

Overall, these findings underscore the multidimensional structure of students’ experiences, illustrating how specific thematic domains co-occur with distinct facets of psychological well-being, academic functioning and performance.

## 4. Discussion

This study examined the academic well-being of students living away from home and enrolled in STEM programmes at an Italian university, integrating quantitative measures of burnout, engagement, self-efficacy, and academic functioning with qualitative narratives from open-ended survey responses. The sample, composed exclusively of students living away from home, including international students, represents a theoretically relevant subgroup in which academic and non-academic stressors accumulate, making both structural and relational vulnerabilities particularly visible. Findings are interpreted within the JD-R framework [8].

The most fundamental finding of this study is that, for students who have left their home environment, academic and non-academic domains are not separable: they form a combined system of demands. Qualitative findings revealed that Organisational practical difficulties (*n* = 412) and Learning-related concerns (*n* = 399) were the most frequently narrated categories, reflecting the structural demands of STEM curricula and the additional daily management burden faced by these students. Unlike commuter students, who can rely on local support for time management, meals, housing, and emotional regulation, non-commuter students must self-organise all aspects of their lives while simultaneously facing intensive academic workloads [20,21].

This dual burden is well captured by the JD-R model [8], in which the depletion of psychological resources occurs when demands—both academic and non-academic—exceed the individual’s coping capacity. In STEM contexts, where cumulative assessment systems, compressed examination periods, complex coursework, and high-performance expectations are the norm [2,3], the additional strain of relocation may push non-commuter students into chronic resource depletion. The co-occurrence analyses confirmed this interpretation: Organisational practical difficulties, Learning, Teaching, and Psychological Impact formed a densely interconnected core, suggesting that relocated STEM students experience these pressures not as distinct problems but as a unified, mutually reinforcing experience of strain. Consistent with Lesener et al. [9], cumulative demands in high-intensity educational environments interact synergistically to predict burnout and disengagement.

The category of Social relationships (*n* = 53), though smaller, was meaningfully interconnected with this core, and social isolation alone accounted for 27 quotations. This finding reflects what Worsley et al. [21] described as the ‘betwixt space’ of the early university transition, in which students who have relocated find themselves detached from established support networks before new bonds are formed. For STEM students living away from home, who must navigate a demanding academic environment while simultaneously rebuilding their social lives from scratch, this transitional vulnerability can be prolonged and costly. Recent longitudinal evidence confirms that loneliness is a significant mediating variable between university transition difficulties and mental health outcomes, and that students who relocate are structurally more exposed to it than commuter peers [22,23].

Among all content categories, Teaching emerged as the most consistent and robust correlate across all psychological and academic outcomes. For STEM students living away from home, the quality of their relationship with teaching is not merely an academic preference: it is one of the few institutional resources capable of compensating—or exacerbating—the emotional costs of living away from home. When teaching is perceived as opaque, authoritarian, or unfair, it removes a potential source of belonging and meaning from an already socially thin environment. Conversely, when teaching is supportive and responsive, it can function as a proximal resource that partially compensates for the absence of familial warmth.

The Teaching category was positively associated with Emotional Exhaustion (β > 0) and Cynicism, and negatively associated with Vigor, Dedication, self-efficacy, study program satisfaction, and perceived academic goal attainment. The strongest effects were on Study Program Satisfaction (β = −0.222, *p* < 0.001) and Dedication (β = −0.153, *p* < 0.001), indicating that critical teaching experiences are not only linked to momentary frustration but are also systematically associated with sustained disengagement and diminished confidence in one’s academic path. For students who have made significant personal and often financial sacrifices to relocate and study at a STEM institution, dissatisfaction with teaching may therefore carry a heavier psychological cost than for commuter peers, who can more easily compartmentalise their academic and personal lives.

The qualitative material revealed that assessment practices were the most frequently raised concern within the Teaching category (99 occurrences), including perceived opacity of grading criteria, limited exam sessions, and a sense of arbitrariness or injustice in evaluation. This finding is consistent with research identifying assessment density and perceived unfairness as among the most potent predictors of burnout in STEM programmes [6,24]. The concentration of assessments into narrow time windows—a structural feature of many Italian STEM universities—amplifies the demand-resource imbalance for students living away from home, who cannot rely on family-based logistical and emotional support during high-stakes examination periods. Introductory reforms such as mid-term examinations or distributed assessment schedules could therefore represent particularly high-impact interventions for this population.

Beyond assessment, students also reported widespread dissatisfaction with pedagogical approaches (93 occurrences), including perceptions of rigid, non-interactive, or insufficiently clear instruction. This is consistent with the literature on autonomy-supportive teaching, which shows that responsive, transparent, and student-centred instruction fosters engagement and reduces burnout [25,26,27]. For students living away from home, who may lack peer study networks and informal academic support in their new environment, the quality of in-class instruction takes on additional importance as a primary source of academic scaffolding.

Despite the prevalence of difficulties, the Positive feelings category—comprising narratives of satisfaction, gratitude, and meaningful learning experiences—demonstrated a robust opposite pattern to Teaching-related narratives. Positive experiences were negatively associated with burnout and positively associated with engagement, self-efficacy, and academic functioning, with the strongest association emerging for Vigor (β = 0.233, *p* < 0.001). This finding underscores that, even within the demanding and socially challenging context of a STEM university for students living away from home, resource-generating processes remain operative and potentially protective.

Within the JD-R framework, these results support the view that positive institutional and pedagogical experiences constitute genuine resources capable of buffering the depletion caused by academic and non-academic demands [5,28]. For students living away from home, who have fewer informal social and emotional buffers available, encounters with supportive professors, meaningful learning experiences, or a sense of academic accomplishment may carry particular motivational weight, functioning as restorative events in an otherwise depleting environment. Conservation of resources theory [29] further supports this interpretation, suggesting that resource gains are especially valuable—and experienced more intensely—in contexts of resource scarcity.

Institutional strategies that intentionally cultivate positive learning experiences—such as faculty development focused on student recognition, mentoring programmes, and spaces for peer collaboration—may therefore yield meaningful well-being benefits specifically for students living away from home. These students, who have invested substantially in their educational migration and are navigating a new environment, may be particularly responsive to experiences of academic validation and belonging within the institution. This is in line with recent meta-analytic evidence indicating that institutional and pedagogical interventions can play a key role in reducing student burnout and improving well-being outcomes [30].

Learning-related category scores were positively associated with emotional exhaustion and cynicism, and negatively associated with dedication and self-efficacy—a pattern consistent with the view that academic overload and structural rigidity function as chronic demands that may contribute to the depletion of motivational and cognitive resources [5,31]. In the qualitative data, excessive academic workload and the lack of exam sessions emerged as the two most structurally salient codes within this category, with students describing a pace of academic demands incompatible with sustainable functioning. For STEM students living away from home, this burden is compounded by the time and energy required to manage daily life independently: cooking, cleaning, commuting, managing finances, and navigating healthcare in an unfamiliar city—tasks that commuter students typically share with their families [20].

The Organisational practical difficulties category (*n* = 412) was the largest, covering study spaces, canteen services, time management, housing, and administrative issues. These often-overlooked aspects of university life accumulate into a constant source of stress for students who rely entirely on the institution and the city. Its co-occurrence with Learning (*n* = 172), Psychological impact (*n* = 86), and Teaching (*n* = 66) shows that organisational problems amplify academic strain: lacking study spaces, clear exam information, or administrative support creates a heavier and qualitatively different academic burden.

Psychological impact narratives (*n* = 175) were linked to higher emotional exhaustion and cynicism, and lower study satisfaction. Reports of stress, anxiety, depression, and even suicidal ideation indicate that for some STEM students living away from home, distress reaches clinical levels. This aligns with evidence that relocated students face higher mental health risks [4,32]. The strong overlap with Psychological impact and Learning (*n* = 121) highlights how academic pressure and emotional distress are deeply interconnected, suggesting the need for integrated institutional responses.

International students showed a distinct pattern. In the co-occurrence network, their difficulties clustered closely with Organisational practical difficulties, Learning, Teaching, and Psychological impact, indicating that their challenges are embedded in the same system, but with added complexity.

Quantitatively, international students’ difficulties were positively associated with Vigor (β = 0.081, *p* < 0.05) but negatively with Perceived Academic Goal Attainment (β = −0.098, *p* < 0.01). This suggests a gap between high motivation and limited perceived progress: despite strong engagement, structural barriers hinder their sense of achievement, consistent with literature describing them as highly motivated but disadvantaged [33,34,35].

Qualitative data highlighted key barriers: language difficulties, bureaucratic complexity, limited access to affordable housing (often requiring an Italian guarantor), social isolation, discrimination, and lack of culturally familiar support. These issues are intensified in the Italian STEM context, where teaching and administration are often Italian-dominant and bureaucratic systems are complex, especially for non-EU students. These findings align with cross-national literature on international students [10,36,37,38].

Co-occurrence analysis showed that social isolation is particularly central for international students. This aligns with prior findings identifying loneliness as a primary challenge, only partially mitigated by group interventions [39]. Overall, this points to the need for targeted, culturally sensitive, and accessible support, rather than generic orientation activities.

Gender issues (*n* = 21) emerged as qualitatively important, with female students reporting marginalisation, interruptions, gendered comments, and occasional harassment. These experiences reflect the well-documented “chilly climate” in STEM [11,12]. For women living away from home, such dynamics may have a stronger psychological impact due to weaker local support networks.

However, no significant gender differences emerged in quantitative outcomes such as burnout or engagement. This may be due to generally high stress levels masking differences, the relatively low proportion of women in the sample, or the fact that gendered experiences shape meaning and identity more than aggregate scores. Mixed-methods approaches are therefore crucial to capture these nuances.

Academic level was a consistent predictor: Bachelor’s students reported lower self-efficacy (β = −0.121, *p* < 0.01) and lower perceived goal attainment (β = −0.161, *p* < 0.001) than Master’s students. This reflects the greater difficulty of early university stages, where academic demands coincide with major social and environmental adaptation challenges.

In the Bachelor subgroup, Social relationships were more central and Economic concerns more prominent than in other groups. This suggests that relational isolation and financial strain are especially salient for undergraduate students living away from home, alongside academic pressure. These findings align with developmental frameworks that view early university as a critical period of social reorganisation [21,40].

Methodologically, the study highlights the value of combining qualitative analysis with quantitative modelling. Narrative-derived category scores explained additional variance beyond sociodemographics (ΔR^2^ = 0.054–0.107), especially for Study Program Satisfaction and Vigor. Integrating coded narratives into a mixed-methods design provides a replicable framework for institutional monitoring, helping universities identify key experience domains linked to psychological outcomes and design targeted interventions [41,42].

### Limitations

The cross-sectional design of the study precludes causal inference, and the observed relationships should therefore be interpreted as associative rather than causal. Although the JD-R framework provides a basis for understanding potential directional processes, the present findings do not allow conclusions about causality or temporal ordering.

Additional limitations should be acknowledged. Data were collected from a single Italian STEM university, limiting generalizability. The quantizing process, while analytically productive, inevitably reduces narrative complexity. Furthermore, the gender imbalance and potential self-selection bias may have influenced the findings. The analytic sample represents a subset of the initially invited population, as participation was voluntary and only a proportion of respondents completed the full survey including the open-ended section. This reduction from the initial invited sample to the final analyzed group may have resulted in a non-random selection of participants.

It is therefore possible that students who completed the full survey differ systematically from non-responders, for example in terms of engagement, motivation, or level of distress. Accordingly, the findings should be interpreted with caution, as they may not fully capture the experiences of the broader student population.

Future longitudinal and intervention-based studies are needed to examine the directionality of these associations and to test whether institutional modifications are associated with improvements in student well-being.

## 5. Conclusions

This study presents an integrated analysis of academic well-being among students living away from home and enrolled in STEM programmes at an Italian university, linking narrated experiences with validated indicators of burnout, engagement, and academic functioning. The findings show that psychological outcomes in this population are shaped not only by individual characteristics but, crucially, by the structural and relational features of the institutional environment, in line with the Job Demands–Resources (JD-R) framework, which conceptualizes well-being as the result of the interplay between demands and resources embedded in the educational context.

Students living away from home in STEM contexts face a complex ecology of demands, where intensive curricula intersect with the logistical, social, and emotional costs of relocation. Teaching quality and assessment practices emerged as the most consistent institutional predictors of burnout and disengagement. For these students, supportive and transparent teaching may serve as a key source of belonging and motivational support, whereas opaque or rigid practices may increase strain. Conversely, positive academic experiences act as meaningful resources that sustain engagement even in high-demand contexts, reinforcing the role of institutional and pedagogical resources in promoting student well-being.

Organisational difficulties—including limited study spaces, administrative inefficiencies, and rigid exam scheduling—were the most frequently reported concerns, underscoring the central role of institutional infrastructure in daily functioning for students without proximal family support. Social isolation was closely linked to learning challenges and psychological distress, highlighting that relational dimensions are integral to academic well-being rather than peripheral.

International students represent a particularly vulnerable subgroup, facing additional barriers related to language, bureaucracy, discrimination, and cultural adaptation. Their profile of sustained engagement alongside lower perceived academic progress calls for culturally sensitive and structurally accessible support strategies.

Overall, the findings highlight modifiable institutional and relational determinants of student well-being. Interventions targeting transparent assessment, autonomy-supportive teaching, distributed exam schedules, functional study and social spaces, and structured peer and international support programmes may yield meaningful benefits.

Importantly, these findings also align with a growing body of research showing that the quality of student–faculty relationships represents not only a determinant of student well-being but also a key resource for the occupational well-being of university staff. Within the JD-R framework, interactions with students can function as both demands and resources: while excessive or inappropriate requests may contribute to workload and burnout, supportive and respectful interactions can enhance work meaning and sustain engagement among academics [43,44]. More broadly, relational and institutional resources have been identified as central drivers of engagement and protective factors against burnout in higher education contexts [45].

Taken together, these findings point to a reciprocal dynamic in which relational quality within the educational environment simultaneously supports both student and faculty well-being. This suggests that student well-being should not be conceptualized as an individual outcome alone, but as part of a broader, interdependent system shaped by institutional design, teaching practices, and the quality of everyday academic relationships. By integrating qualitative narratives with quantitative indicators, this study offers a framework for institution-level mental health promotion tailored to the specific ecology of students living away from home in STEM environments, and contributes to the growing literature advocating for structurally embedded and relationally grounded approaches to well-being in higher education.

## Figures and Tables

**Table 1:** Content Categories and Number of Occurrences Identified in Students’ Responses.

Content Categories	Occurrences (*N*)
Communication problems	17
Economic concerns	24
Future work concerns	14
Health concerns	11
Gender issues	21
International students’ difficulties	29
Learning	399
Teaching	206
Organisational practical difficulties	412
Special needs	5
Psychological impact	175
Social relationships	53
Positive feelings	28

**Table 2:** Descriptive statistics, reliability, and intercorrelations among study variables.

Variable	M	SD	α	ω	1	2	3	4	5	6	7
1. EE	18.91	6.77	0.880	0.914	-						
2. CYN	10.49	7.19	0.889	0.923	0.571 ***	-					
3. VIG	11.48	5.97	0.840	0.895	−0.445 ***	−0.358 ***	-				
4. DED	18.43	7.38	0.931	0.948	−0.398 ***	−0.645 ***	0.508 ***	-			
5. S-E	17.82	6.25	0.885	0.908	−0.486 ***	−0.444 ***	0.491 ***	0.511 ***	-		
6. SPS	5.71	2.38	-	-	−0.469 ***	−0.549 ***	0.440 ***	0.567 ***	0.546 ***	-	
7. PAGA	55.73	23.82	-	-	−0.305 ***	−0.281 ***	0.299 ***	0.284 ***	0.463 ***	0.543 ***	-

Note. EE = Emotional Exhaustion, CYN = Cynicism, VIG = Vigor, DED = Dedication, S-E = Self-Efficacy, SPS = Study Program Satisfaction, PAGA = Perceived Academic Goal Attainment. *** *p* < 0.001.

**Table 3:** Hierarchical regression analyses predicting psychological outcomes from content categories (standardized coefficients, full model).

	EE	CYN		DED	S-E	SPS	PAGA
Variables and Categories	Emotional Exhaustion	Cynism	Vigor	Dedication	Self-Efficacy	Study Program Satisfaction	Perceived Academic Goal Attainment
Age	0.081 *	0.139 ***	−0.025	−0.101 **	−0.010	−0.097 **	0.018
Female (1 = yes)	0.041	0.029	−0.008	−0.048	−0.043	−0.047	0.010
Bachelor’s (1 = yes)	−0.018	−0.051	−0.047	−0.059	−0.121 **	−0.109 **	−0.161 ***
Communication	−0.011	−0.012	0.030	−0.008	−0.002	0.053	0.036
Economic/Future	−0.032	−0.043	0.033	0.019	−0.003	−0.004	0.063
Gender Issues	0.033	0.011	−0.038 *	−0.023	−0.022	−0.014	0.002
Health	0.022	0.022	−0.058	0.010	−0.062	−0.006	−0.015
International	0.037	0.011	0.081 *	−0.026	−0.046	−0.004	−0.098 **
Learning	0.092 *	0.094 *	−0.020	−0.074 *	−0.099 **	−0.034	−0.021
Organizational	0.024	−0.062	−0.005	0.017	0.037	0.012	0.001
Psychol. Impact	0.120 ***	0.083 *	−0.055	−0.045	−0.040	−0.077 *	−0.026
Social	−0.041	0.019	0.040	0.020	0.043	0.033	0.017
Teaching	0.104 **	0.111 **	−0.110 **	−0.153 ***	−0.125 ***	−0.222 ***	−0.166 ***
Positive	−0.080 *	−0.055	0.233 ***	0.117 **	0.149 ***	0.162 ***	0.134 ***
R^2^	0.073	0.077	0.096	0.063	0.099	0.131	0.096
ΔR^2^	0.061	0.056	0.091	0.054	0.074	0.107	0.062

Note. Full models include age, gender (female = 1), and academic level (Bachelor’s = 1) as covariates. ΔR^2^ represents the additional variance explained by content categories beyond sociodemographic factors. * *p* < 0.05, ** *p* < 0.01, *** *p* < 0.001.

## Data Availability

The data presented in this study are available on reasonable request from the corresponding author. The data are not publicly available due to privacy and confidentiality considerations related to the inclusion of open-ended responses.
